# Plans for Churches

**Published:** 1858-07

**Authors:** 


### "PLANS FOR CHURCHES"

Is the title of a book just published in New York, price Ten Dollars—if it is worth ten cents, we will acknowledge ourselves mistaken. We do not believe there is a properly built church in the city—a church, healthful, safe, and best adapted to its purposes; most of them leak, and hence have damp, mouldy, rotting walls and plastering; very few of them are constructed for thorough ventilation; not one in twenty allows the minister to speak with comfort to himself; and although few of them hold over a thousand persons comfortably, yet those who occupy the seats distant from the speaker can only hear by painful attention.

It was well said by one of our best clergymen: "The Adversary has a hand in building our places of worship." And worse than anything yet mentioned, is the want of proper facilities for egress, in case of sudden alarm. They are generally constructed so that the whole congregation, from the body of the house, and from the galleries, are poured into a vestibule, not ten feet broad, with three or four pairs of doors, some opening inwards, others sliding, *not one outwards!* In the event of an alarm by fire, or hurricane, or by any accident occasioning a panic, the crushing and suffocation of numbers would be inevitable. It ought to be compulsory, that every church should have at least two large folding-doors in each end of the building, *opening outwards.* But this will not be done until fifty or a hundred persons are murdered; and then the newspapers and the public will suddenly grow wise and indignant—as in the maiming and destruction of children in the public school buildings, in several instances, yet fresh in our memories. In New York City, there are several hundred churches, not one single one of which, to the best of our knowledge, is constructed according to the dictates of the commonest kind of common sense. If the author of the "*Plans for Churches*" was the builder of any one of these, he is clearly unfit to write a book on the subject; if he never built a church, then he knows nothing about church-building, practically.

It is a growing fashion, conspicuous in the more recently erected houses of public worship, to be lavish in gew-gaw ornaments, with all the colors of the rainbow, of the most glaring sort—yellow, red, and blue; possibly looking to the time when they may be occasionally "let" for more worldly purposes—not an uncommon thing even now, in spite of the very practical and plain conduct of the MASTER, when he whipped the thieving money-changers out of his FATHER'S house. In our opinion, a building consecrated to Divine worship ought not to be used for any other purpose whatever; not even for the meeting of church judicatories—we, perhaps, had better have said, "*least* of all, for church judicatories"—remarkable sometimes for shameful personalities and bitter recriminations. Let the house of GOD and his worship have no other associations in the minds of our children than those of serenity, of calm devotion, and of reverential awe. To this end, let our "*church plans*" cultivate plainness, capacity, healthfulness, and safety; and not least, let them seek to attain that shape and arrangement which will contribute most to the ease of both speaker and hearer: the want of this has prematurely laid aside many a useful clergyman; for, to speak with safety to the vocal organs and lungs, a man should be conscious of no effort in relation to those parts if he is conscious of that effort, every sentence uttered is an injury.

